# A Simple Cable Method for Intraoperative Limb Alignment Assessment

**Published:** 2016-03

**Authors:** CK Chan, R Shanmugam

**Affiliations:** Department of Orthopaedic Surgery (NOCERAL), University Malaya Medical Centre, Kuala Lumpur, Malaysia

Achieving good mechanical axis of the lower extremity is of paramount importance in orthopaedic surgery, not only during joint replacement but also in fracture fixation and deformity correction. However, it is difficult to determine the mechanical axis during surgery. Many techniques have been proposed in clinical practice such as visual inspection, cable method, alignment rods, the axis board^1^, panoramic radiograph images^2^ and navigation systems. Among all, cable method is proven to be one of the practical and uncomplicated way yet valid enough to control the mechanical axis intra-operatively^3^.

The Limb Lengthening and Reconstruction Surgery (LLRS) unit of University Malaya Medical Centre devised a cable method to assess the mechanical axis alignment intraoperatively. Our apparatus includes a 1.5mm stainless steel cable, 2 aluminum hooks, 2 wooden bars, weight holder and weight (we used a 500 mls saline drip bottle in this setting) (Fig 1a). Aluminum plate is chosen as it is malleable and easily machined to desirable shapes. One end of the plate is bent so that it can be hooked onto the operating table side bars (Fig 1b). On the other end a hole with extension slot is created for attachment of the wooden bar (Fig 1a). Around the middle of the plate, we use a bolt and nut to serve as stopper to prevent tilting movement due to the pulling effect of the cable (Fig 1b).

**Fig. 1a:**
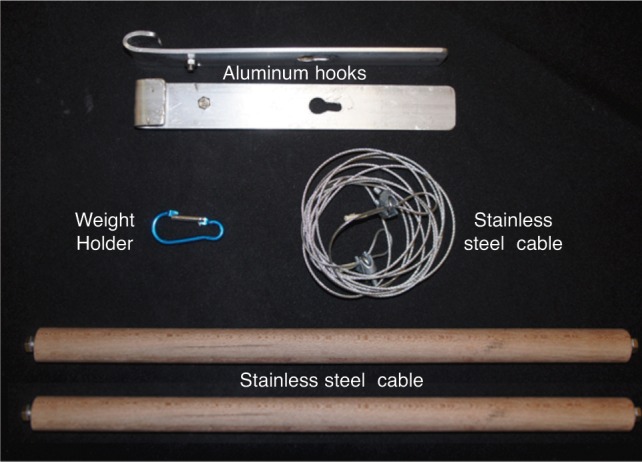
This photograph shows the main components of limb alignment device.

**Fig. 1b:**
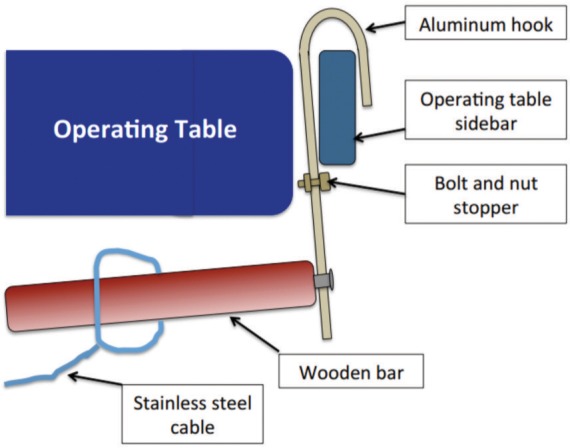
This drawing shows how the alignment device was placed onto the side bar of operating table.

Our apparatus can be installed under the operating table before surgery, and the position of the cable can be further adjusted during surgery. The proximal and distal end of the cable is positioned at the centre of the femoral head and ankle respectively and confirmed by intensifier imaging (Figure 2a-c). This simple cable method is cost effectiveness, simple to set up and easy to reproduce. We believe this apparatus is of substantial value in checking the coronal plane alignment during orthopaedic procedures of the lower limbs.

**Fig. 2a:**
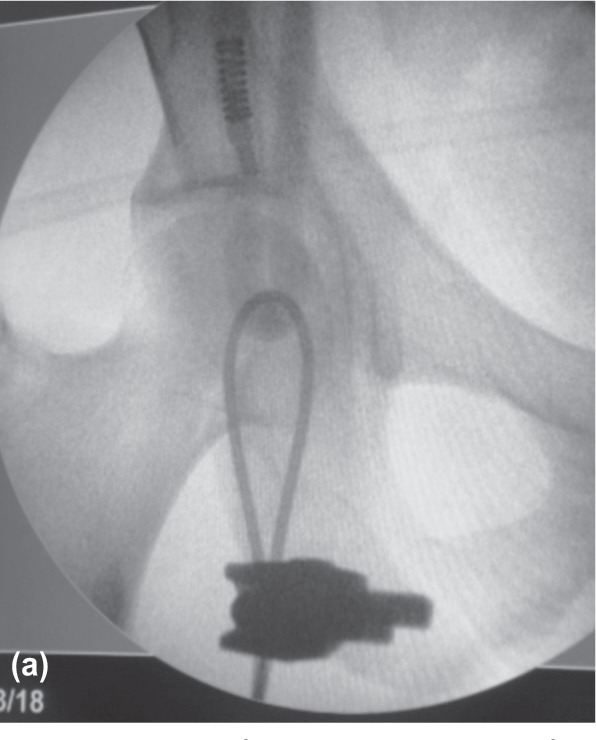
Proximal wire was positioned at the centre of femoral head.

**Fig. 2b:**
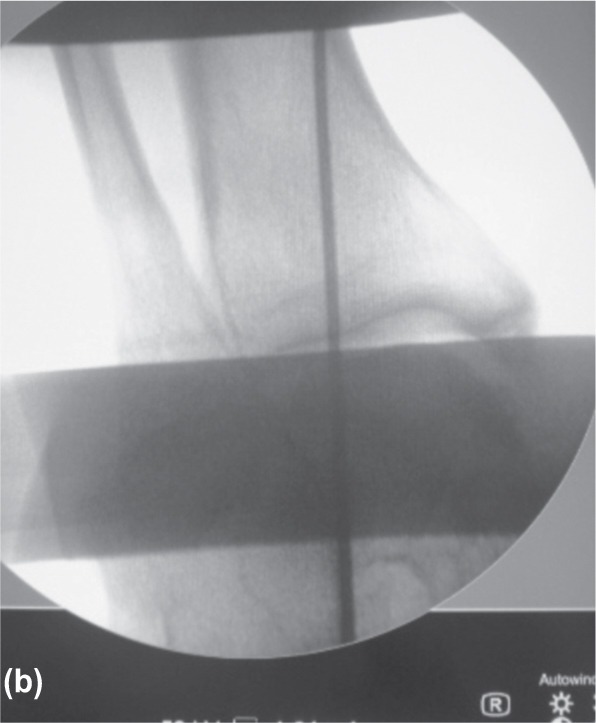
Distal wire was adjusted until the wire passed the centre of ankle / talus.

**Fig. 2c:**
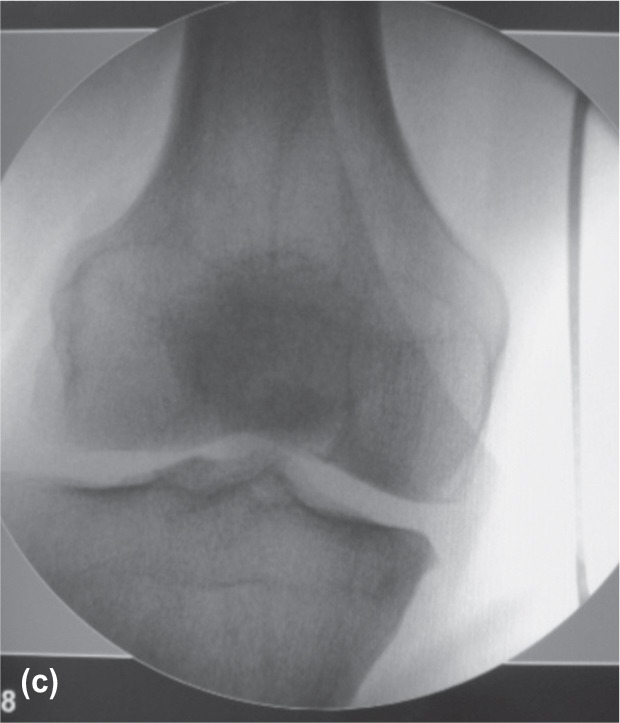
At the knee level, position of he wire indicated medial deviation of the lower limb mechanical axis.
